# Contrast-Enhanced Endoscopic Ultrasound Detects Early Therapy Response Following Anti-TNF Therapy in Patients with Ulcerative Colitis

**DOI:** 10.1093/ecco-jcc/jjae034

**Published:** 2024-03-08

**Authors:** Mark Ellrichmann, Berenice Schulte, Claudio C Conrad, Stephan Schoch, Johannes Bethge, Marcus Seeger, Robert Huber, Madita Goeb, Alexander Arlt, Susanna Nikolaus, Christoph Röcken, Stefan Schreiber

**Affiliations:** Interdisciplinary Endoscopy, Medical Department I, University Hospital Schleswig-Holstein, Campus Kiel, Kiel, Germany; Interdisciplinary Endoscopy, Medical Department I, University Hospital Schleswig-Holstein, Campus Kiel, Kiel, Germany; Interdisciplinary Endoscopy, Medical Department I, University Hospital Schleswig-Holstein, Campus Kiel, Kiel, Germany; Interdisciplinary Endoscopy, Medical Department I, University Hospital Schleswig-Holstein, Campus Kiel, Kiel, Germany; Interdisciplinary Endoscopy, Medical Department I, University Hospital Schleswig-Holstein, Campus Kiel, Kiel, Germany; Interdisciplinary Endoscopy, Medical Department I, University Hospital Schleswig-Holstein, Campus Kiel, Kiel, Germany; Institute of Biomedical Optics, University of Luebeck, Luebeck, Germany; Department of Internal Medicine, Israelitic Hospital Hamburg, Hamburg, Germany; Department of Internal Medicine, Israelitic Hospital Hamburg, Hamburg, Germany; Interdisciplinary Endoscopy, Medical Department I, University Hospital Schleswig-Holstein, Campus Kiel, Kiel, Germany; Institute of Pathology, University Hospital Schleswig-Holstein, Campus Kiel, Kiel, Germany; Interdisciplinary Endoscopy, Medical Department I, University Hospital Schleswig-Holstein, Campus Kiel, Kiel, Germany

**Keywords:** Ulcerative colitis, biological therapy, endoscopic ultrasound

## Abstract

**Background and Aims:**

Though colonoscopy plays a crucial role in assessing active ulcerative colitis [aUC], its scope is limited to the mucosal surface. Endoscopic ultrasound [EUS] coupled with contrast-enhancement [dCEUS] can precisely quantify bowel wall thickness and microvascular circulation, potentially enabling the quantitative evaluation of inflammation. We conducted a prospective, longitudinal study to assess therapy response using dCEUS in aUC patients undergoing treatment with adalimumab [ADA] or infliximab [IFX].

**Methods:**

Thirty ADA- and 15 IFX-treated aUC patients were examined at baseline and at 2, 6, and 14 weeks of therapy and 48 weeks of follow-up. Bowel wall thickness [BWT] was measured by EUS in the rectum. Vascularity was quantified by dCEUS using rise time [RT] and time to peak [TTP]. Therapy response was defined after 14 weeks using the Mayo Score.

**Results:**

Patients with aUC displayed a mean BWT of 3.9 ± 0.9 mm. In case of response to ADA/IFX a significant reduction in BWT was observed after 2 weeks [*p* = 0.04], whereas non-responders displayed no significant changes. The TTP was notably accelerated at baseline and significantly normalized by week 2 in responders [*p* = 0.001], while non-responders exhibited no significant alterations [*p* = 0.9]. At week 2, the endoscopic Mayo score did not exhibit any changes, thus failing to predict treatment responses.

**Conclusion:**

dCEUS enables the early detection of therapy response in patients with aUC, which serves as a predictive marker for long-term clinical success. Therefore, dCEUS serves as a diagnostic tool for assessing the probability of future therapy success.

## 1. Introduction

Ulcerative colitis [UC] is a chronic disorder characterized by a relapsing and remitting course, with the potential for progression from mild, asymptomatic to extensive colonic inflammation. This progression gives rise to clinical manifestations such as recurrent bloody stools, impaired colonic motility, the risk of permanent fibrosis and tissue damage, systemic symptoms, and, in some cases, necessitates surgical intervention. Over a 10-year period, ~31% of patients initially diagnosed with limited UC will experience disease extension, and colectomy may be necessary in 10–15% of UC cases. Achieving short-term disease control to alleviate symptoms and prevent long-term complications are of paramount significance when devising individualized treatment strategies for patients with inflammatory bowel disease [IBD].^1^

The STRIDE [Selecting Therapeutic Targets in Inflammatory Bowel Disease] II initiative offers a comprehensive set of evidence- and consensus-based recommendations for implementing treat-to-target strategies in both adult and paediatric patients with IBD reaffirming the long-term targets initially outlined in STRIDE-I, which include the attainment of clinical remission and endoscopic healing. In addition to these, STRIDE-II has introduced several new objectives, such as the elimination of disability, restoration of patients’ quality of life, and the promotion of normal growth in paediatric populations. Moreover, as part of short-term targets, symptomatic relief and the normalization of serum and faecal markers have been identified. Notably, the STRIDE-II initiative initially did not endorse histological and transmural healing as primary treatment targets. However, it did recommend the inclusion of histological healing as a supplementary measure to endoscopic remission to signify a deeper level of healing.^2,3^

However, the effective management of IBD entails the comprehensive evaluation of various dimensions of healing, comprising mucosal healing, histological healing, and transmural healing.

Endoscopic assessment of mucosal healing is a fundamental component of evaluating disease severity and has emerged as a pivotal prognostic factor that can forecast the maintenance of clinical remission and the avoidance of surgical resection.^4–6^ The most widely used metrics for evaluating endoscopic disease activity include the UC Endoscopic Index of Severity [UCEIS] and the endoscopic Mayo score. Though these scores have been validated for the initial evaluation of inflammation levels in UC, they have not attained comprehensive validation for assessing early therapeutic response and remain susceptible to significant interobserver variability.^7–10^ The evaluation of mucosal healing serves as a diagnostic tool after the induction phase, typically conducted 6–8 weeks following the initiation of therapy. However, limited attention has been devoted to the concept of early mucosal healing within the initial 4 weeks of treatment. Furthermore, a significant drawback of this approach is the exclusive assessment of the mucosal surface, without considering the potential alterations in deeper layers of the gastrointestinal wall and the dynamic changes in mucosal and submucosal vascularity, which are pertinent to the disease process.

By definition, Crohn’s disease [CD] has a transmural involvement of the gastrointestinal tract. In this regard, transmural healing in CD represents a critical and evolving concept in the clinical management of this chronic inflammatory condition. It pertains to the comprehensive healing of all layers of the intestinal wall, extending beyond mere mucosal resolution. Achieving transmural healing signifies the complete restoration of the structural and functional integrity of the gastrointestinal tract, including the submucosal and serosal layers. This deep healing is of paramount importance as it significantly correlates with long-term outcomes, minimizing the risk of complications such as strictures, fistulas, and abscesses. Mucosal and submucosal healing in UC, although less commonly discussed and evaluated, is also emerging as an important aspect of clinical management.

A recent study demonstrated that mucosal and submucosal thickening, as assessed by intestinal, transabdominal ultrasound [tUS], is of superior predictive value for colectomy risk in patients with UC when compared to endoscopic evaluation.^11^ This highlights the potential importance of colonic wall assessments in clinical decision-making for UC management.

In a pilot study by our group, we evaluated the utility of high-definition endoscopic ultrasound [EUS] in patients with IBD. Our findings revealed a strong correlation between transmural EUS measurements of colonic wall thickness and histological evidence of inflammation,^12^ which is consistent with early results reported by Shimizu *et al*.^13^

Based on these compelling data and the emerging role of deep healing in UC, EUS of the colonic wall holds promise as a valuable tool for the precise evaluation of mucosal and submucosal inflammation levels and the prediction of both early and long-term responses to biological therapies. Therefore, this study was conducted to evaluate the potential role of dynamic contrast-enhanced EUS [dCEUS] of the colon in patients with active UC undergoing treatment with anti-TNF-alpha antibodies [anti-TNF], specifically adalimumab [ADA] or infliximab [IFX], for the early detection of therapeutic responses in relation to long-term follow-up assessment.

## 2. Patients and methods

### 2.1. Study design

In this prospective, longitudinal cohort study, individuals with active UC were enrolled for radial EUS evaluations of the rectum and sigmoid. These assessments were conducted prior to treatment initiation and subsequently at 2, 6, and 14 weeks following the commencement of therapy, utilizing either ADA or IFX, in accordance with current treatment protocols. Patients demonstrating a favourable response to the therapeutic intervention proceeded into a long-term follow-up phase, wherein they underwent re-evaluation via sigmoidoscopy and rectal EUS at 48 weeks post-treatment initiation, followed by ongoing clinical observation.

Patient recruitment and the selection of therapeutic agents were conducted independently by our outpatient department. Radial EUS was employed to measure mucosal, submucosal, and bowel wall thickness [BWT] in the distal sigmoid and rectum. Subsequently, the vascularity of the intestinal wall was assessed using dCEUS following the injection of 2.5 mL of the contrast agent SonoVue. Contrast kinetics were quantified in terms of rise time [RT] and time to peak intensity [TTP]. EUS findings were juxtaposed with data acquired from healthy controls [HC] who underwent screening colonoscopy, as well as individuals with UC in remission [rUC] undergoing surveillance colonoscopy. Data from EUS examinations across all groups were correlated with the endoscopic Mayo score and histological inflammation scores derived from biopsies obtained from the same regions evaluated via EUS. Response to therapy was defined as clinical remission at week 14 and was subsequently compared to endoscopic and histological outcomes. Furthermore, in the subgroup of patients EUS data were compared to tUS images.

The study was conducted in a prospective, comparative manner in accordance with the ethical principles of the *Declaration of Helsinki*. Ethical permission was granted by the Ethics Committee of the Christian-Albrechts-University Kiel, Kiel, Germany [reference number: A104/13]. The study was licensed at ClinTrials.gov [licence number NCT02694588]. Written informed consent for the procedure and the inclusion into the study was obtained from all patients and control subjects.

### 2.2. Study aims, hypothesis, and endpoints

The objective of this investigation was to assess the role of mucosal and submucosal healing, as determined by BWT and vascularity, in patients with active UC undergoing treatment with ADA or IFX for the early detection of therapeutic response. Our hypothesis was centred on the idea that alterations in BWT precede mucosal healing within the initial 2 weeks of therapy, offering a means to predict treatment response during this early phase.

The primary endpoint of this study was defined as the assessment of BWT in patients with active UC following 2 weeks of biological therapy. Secondary endpoints encompassed the dynamic alterations in vascularity, evaluated via TTP, variations in the endoscopic Mayo score in correlation with histological inflammation scores, as well as differences in BWT as assessed by tUS. Additionally, differences in BWT between the patient groups, clinical symptoms, UC-related complications, and their interplay were investigated as part of the secondary endpoints.

### 2.3. Patients

Patients between 18 and 75 years of age with active UC, defined as an endoscopic subscore of ≥2 with involvement of the sigmoid colon and rectum were eligible to participate in this study.^14^ Additional inclusion and exclusion criteria are summarized in Supplement 1. Age- and sex-matched patient groups with UC in remission as well as patient groups undergoing screening colonoscopy served as controls. The controls [rUC and HC] were recruited through our outpatient clinic and were systematically matched by the study personnel with individuals from the UC population already enrolled at the corresponding time points.

### 2.4. Indication for therapy and definition of remission

The decision for and administration of ADA [Humira; AbbVie] or IFX [Remicade; MSD Sharp Dome] therapy were determined and initiated by an independent gastroenterologist at the outpatient clinic of the Department of Medicine I, University Hospital Schleswig-Holstein, Campus Kiel, or in a private medical practice, in accordance with prevailing therapeutic guidelines. It is essential to note that the decision and execution of therapy were entirely unrelated to the patient recruitment process for this study.

Clinical response was defined as a reduction of at least 3 points from baseline in the total Mayo score, along with a decrease of at least 30% from baseline, following 14 weeks of biological therapy. Clinical remission was characterized by a total Mayo score of less than 2 points, with no individual subscore exceeding 1. Mucosal healing was defined as a Mayo endoscopic subscore of 0 or 1 at the 14-week mark.

Upon achieving clinical and endoscopic responses, patients underwent a re-evaluation through sigmoidoscopy and rectal EUS at 48 weeks following the initiation of therapy. Subsequently, clinical follow-up was initiated in accordance with standard clinical practice guidelines.

### 2.5. Adjunctive therapies

If concurrent corticosteroid therapy was used, a stable dosage [prednisone ≥20 mg/day for at least 2 weeks or <20 mg/day for at least 40 days] was required before initiation of biological therapy. In patients with a satisfactory clinical response, the corticosteroid could be tapered after week 8 at the discretion of the gastroenterologist. Stable dosages over a period of 3 months prior to baseline were required in patients receiving immunomodulators [≥1.5 mg/kg/day or the highest tolerated dosage of azathioprine or ≥1 mg/kg/day or the highest tolerated dosage of 6-mercaptopurine with stable dosage for ≥1 month prior to baseline]. Concurrent immunomodulator dosages remained constant during study treatment. Rectal therapies with mesalamine or glucocorticoids had to be stopped at least 2 weeks before index examination.

### 2.6. Sigmoidoscopy

Preparation for sigmoidoscopy was ensured by transanal, retrograde irrigation with 130 mL of Natriumdihydrogenphosphat [Klistier, Fresenius Kabi Deutschland] ~15 min prior to endoscopy. Endoscopic procedures were carried out under conscious sedation using Propofol [Propofol-Lipuro 10 mg/mL, Braun Melsungen] at the patient’s request, while each patient was monitored throughout the procedure according to current guidelines.^15^

### 2.7. Mayo endoscopic subscore

During sigmoidoscopy macroscopic findings were categorized according to the endoscopic Mayo score [eMayo]. This consists of a four-stage grading system [from 0 to 3]: 0, normal or inactive disease; 1, mild disease: erythema, decreased vascular pattern, mild friability; 2, moderate disease: marked erythema, absent vascular pattern, friability, erosions; 3, severe disease: ulceration, spontaneous bleeding.

### 2.8. Endoscopic ultrasound

#### 2.8.1. Assessment of BWT

Following sigmoidoscopy, EUS was conducted using a 7-MHz forward-viewing radial echoendoscope [EG-3670URK; Pentax] connected to a Hitachi console [HI Vision Avius; Hitachi]. This setup provided a 360° radial image in a plane perpendicular to the axis of the proximal tipand enabled colour Doppler imaging of vessels. To ensure acoustic coupling, the colon lumen was filled with sterile, de-aerated water. The volume of water installation was standardized to 50 mL to avoid non-physiological distention of the recto-sigmoid colon and therefore avoid potential bias in the evaluation of BWT.

Before measuring BWT, 20 mg of intravenous butylscopolamine [Buscopan 20 mg/1 mL; Boehringer Ingelheim Pharma] was administered to minimize potential bias from colonic peristalsis. BWT measurements were carried out using EUS in three specific locations: the distal sigmoid colon (20 cm above the anus [ab ano]), the rectosigmoid junction [15 cm ab ano], and the middle third of the rectum [10 cm ab ano]. To reduce sampling errors, EUS measurements were repeated three times at each site. Subsequently, a mean BWT was calculated across all three locations for further analysis. BWT was defined as the distance between the proximal and distal hyperechoic wall layers, representing the first mucosal layer and the serosa.

#### 2.8.2. dCEUS

In addition, dCEUS was conducted following the injection of 2.5 mL of sulphur hexafluoride-filled microbubbles into an antecubital vein using SonoVue [8 µL/mL; Bracco International]. Upon initiating the contrast injection, a video loop was recorded. Temporal changes in the intensity of the contrast agent post-injection were subsequently computed using specialized quantification software integrated into the EUS processor [Hitachi], as previously described.^16^

Briefly, TTP was determined as the interval between the onset of contrast injection and the moment of peak enhancement in the echo signals. RT was defined as RT = TTP − TI, where TI represents the instant when the maximum slope tangent intersects the *x*-axis.

#### 2.8.3. Histological evaluation

Following the conclusion of sigmoidoscopy and EUS, biopsies were taken from the sigmoid colon and rectum, targeting the specific regions evaluated during EUS examination. One impartial pathologist [CR], unaware of both the acquired clinical data and the corresponding treatment phase, analysed all biopsy specimens [Department of Pathology, University Hospital Schleswig-Holstein, Campus Kiel, Germany]. The degree of activity was quantified using the Nancy Histological Index for Ulcerative Colitis as described.^17^

#### 2.8.4. Interobserver variability

In a subgroup of 20 patients, rectal EUS and sigmoidoscopies were independently performed by three distinct endoscopists, including two senior practitioners and one trainee. The senior endoscopists possessed extensive experience [with more than 500 rectal EUS procedures and over 2000 colonoscopies], whereas the trainee had limited exposure [fewer than 25 rectal EUS procedures and fewer than 200 colonoscopies]. Interobserver variability was assessed, and respective Kappa values were employed to gauge the level of agreement. These Kappa values were interpreted as follows: Kappa < 0, no agreement; Kappa = 0.00–0.20, slight agreement; Kappa = 0.21–0.40, fair agreement; Kappa = 0.41–0.60, moderate agreement; Kappa = 0.61–0.80, substantial agreement; and Kappa = 0.81–1.00, almost perfect agreement.

#### 2.8.5. Transabdominal, intestinal ultrasound

Furthermore, we assessed the utilization of tUS in direct comparison with dCEUS within a subset of 20 patients possessing comprehensive tUS image datasets obtained at baseline and following a 14-week treatment period. tUS examinations were conducted at anatomically matched sites to dCEUS, specifically the distal sigmoid, rectosigmoid junction, and middle part of the rectum. All tUS procedures were executed by a highly experienced and skilled ultrasonographist [MS]. To mitigate potential bias introduced by intraluminal air during sigmoidoscopy, tUS was conducted immediately preceding the endoscopy.

#### 2.8.6. Statistics

Results are presented as means ± SD [standard deviation] unless otherwise stated. The total and endoscopic Mayo score are displayed as median and interquartile range (median [25/75%]). Differences were calculated using the Mann–Whitney test for unpaired, non-parametric comparisons. The Bonferroni correction for multiple comparisons of data was applied where relevant. Youden’s index *J* [*J* = Sensitivity + Specificity − 1] was used in conjunction with receiver operating characteristic [ROC] analysis to calculate cut-off values. Cohen’s Kappa was applied to estimate interobserver reliability of the eMayo score, whereas differences of the quantitative measurements [BWT, TTP] are expressed as percentage differences of the mean values of two senior observers. Comparison of BWT assessed by dCEUS or tUS was carried out using aWilcoxon test for matched pair but non-parametric analyses and supported by regression analyses. A *p*-value of <0.05 was used to indicate statistical differences. Statistical analyses were carried out using GraphPad Prism, version 9.0 [GraphPad Software].

#### 2.8.7. Sample size calculation

The sample size for this study was determined using G*Power calculation software [version 3.1.9.2, http://gpower.hhu.de]. The estimation process was predicated on the following assumptions: prior investigations conducted by our research team revealed a BWT of 1.5 ± 1.4 mm in HC, whereas patients with active UC exhibited a BWT measuring 3.2 ± 1.7 mm.^12^ Our underlying hypothesis posited that a substantial reduction of ~50% in BWT would be attained following transmural healing consequent to anti-TNF therapy. A significance level [α] of 5% and a statistical power [1 − β] of 80% were set as the parameters for this calculation. Accounting for an anticipated dropout rate of 10%, our final sample size estimation dictated that a total of 50 patients with UC would be required to ensure the study’s statistical power.

## 3. Results

### 3.1. Patients and characteristics

**Figure 1.**
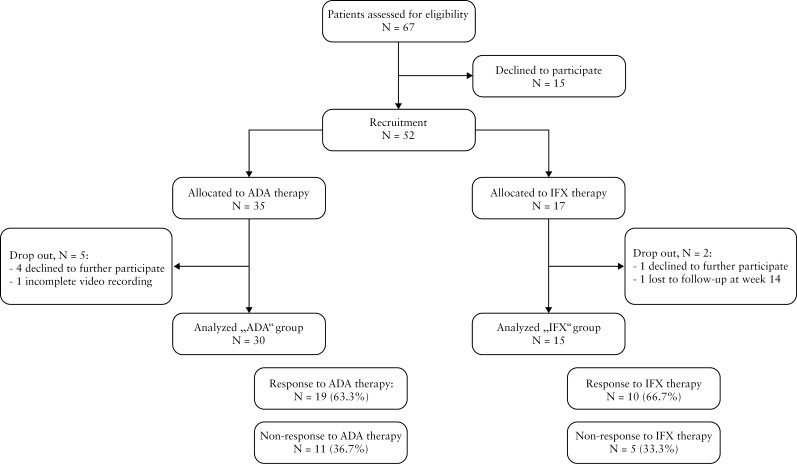
Study overview, patient recruitment, and assessment

A total of 52 patients who underwent anti-TNF therapy were subjected to assessment. Recruitment of the first patient commenced in November 2017 [first patient in], while the final patient successfully completed the endoscopic follow-up period of 48 weeks in December 2020 [last patient out], and clinical long-term follow-up was terminated in January 2022. Within the group treated with ADA, four patients declined to undergo endoscopic examinations at either week 6 or 14. Additionally, one patient had insufficient video recording during application of the contrast agent at week 6 and was therefore not evaluable. In the IFX group, one patient discontinued participation in the study after 5 weeks of treatment, and another patient was lost to follow-up after 14 weeks of treatment [Figure 1].

Of the initial cohort, 30 patients [66.7%] receiving ADA therapy and 15 patients [33.3%] receiving IFX successfully completed the study, providing a comprehensive dataset for analysis [Figure 1]. Between both groups no significant differences were observed in terms of age and sex distribution as well as prior or concurrent medication use or underlying medical conditions. Further details regarding patient characteristics at baseline are presented in Table 1.

**Table 1.** Patient characteristics

Baseline characteristics	Anti-TNF therapy			
	Total	Response	Non response	*p*-value
Number of patients [*N*] [% of total]	45 [100]	29 [64.4]	16 [35.6]	–
Age [years ± SD]	35.0 ± 11.8	35.5 ± 12.7	34.1 ± 10.3	0.8
Female [*N*] [% of total]	23 [51.1]	16 [35.6]	7 [15.5]	0.5
No prior IBD-specific medication [*N*] [% of total]	24 [53.3]	16 [35.6]	8 [17.8]	0.7
Prior IBD-specific medication [*N*] [% of total]	21 [46.7]	13 [28.9]	8 [17.8]	0.7
5-ASA [*N*] [% of total]	12 [26.7]	7 [15.5]	5 [11.1]	0.9
Azathioprine [*N*] [% of total]	12 [26.7]	8 [17.8]	4 [8.9]	0.7
6-Mercaptopurine [*N*] [% of total]	8 [17.8]	4 [8.9]	4 [8.9]	0.6
Stable dose of corticosteroids [*N*] [% of total]	19 [42.2]	11 [24.4]	8 [17.8]	0.5
Active nicotine consumption [*N*] [% of total]	8 [17.8]	6 [13.3]	2 [4.4]	0.7
Total Mayo score (median [IQR])	9 [8–10]	9 [9–10]	8 [8–10]	0.7
Endoscopic Mayo score (median [IQR])	2 [2–3]	2 [2–3]	2 [2–3]	0.9
Nancy histological index (median [IQR])	2 [2–3]	2 [1.75–3]	2 [2–3]	0.7
Faecal calprotectin [µg/g ± SD]	491.5 ± 362.3	510.6 ± 383.9	456.9 ± 328.7	0.8
C-reactive protein [mg/L ± SD]	14.90 ± 14.2	14.1 ± 12.7	39.4 ± 99.0	0.8
Time of follow-up [weeks ± SD]	73.4 ± 11.4	73.3 ± 13.4	73.5 ± 6.8	0.9

*p*-values are expressed for the comparison of response vs non response.

Twenty HC undergoing screening colonoscopy and 20 rUC patients undergoing surveillance colonoscopy served as controls. Age and sex distribution matched the first 20 patients recruited in the UC population.

### 3.2. Bowel wall thickness

**Figure 2.**
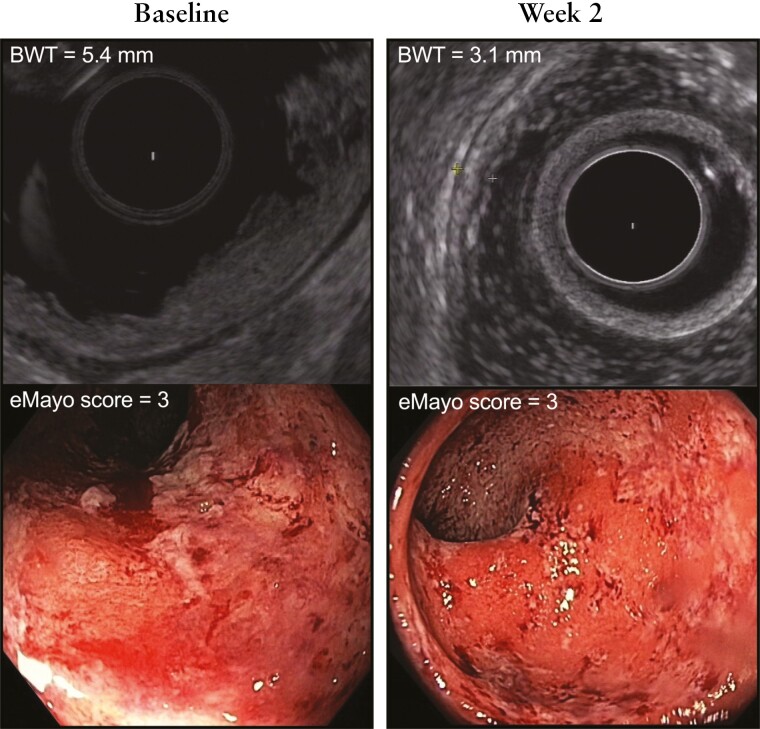
Comparison of rectal EUS assessing bowel wall thickness [BWT] and endoscopic Mayo score at baseline and week 2 in a representative patient undergoing treatment with adalimumab and responding to therapy.

Patients undergoing biological therapy exhibited a BWT of 3.8 ± 1.3 mm at baseline assessment. Within the initial 2 weeks of treatment, a substantial reduction in BWT to 3.1 ± 1.1 mm was observed in patients demonstrating a positive therapeutic response [*p* = 0.008 compared to baseline], while BWT remained unchanged at 4.1 ± 1.3 mm in those exhibiting no response. Remarkably, even at this early stage of treatment, a significant distinction in BWT was evident between responsive and non-responsive patients. As therapy progressed, BWT continued to diminish, measuring 2.6 ± 0.9 mm after 6 weeks and 2.3 ± 0.6 mm after 14 weeks. Conversely, in non-responsive patients, BWT remained unaltered throughout the entire treatment duration [non-response: BWT_week6_ = 3.9 ± 1.3 mm; BWT_week14_ = 3.8 ± 1.0 mm]. No discernible differences were noted when comparing the two distinct treatment regimens [Figure 2; Table 2].

**Table 2.** Summary of inflammatory indicators throughout the course of therapy from baseline to week 14.

Anti-TNF-alpha	Total Mayo score (median [IQR])			Endoscopic Mayo score (median [IQR])			Nancy histology index (median [IQR])			Bowel wall thickness [mm] [mean ± SD]			Rise time [s] [mean ± SD]			Time to peak [s] [mean ± SD]		
	Response	Non-response	*p*-value	Response	Non-response	*p*-value	Response	Non-response	*p*-value	Response	Non-response	*p*-value	Response	Non-response	*p*-value	Response	Non-response	*p*-value
Baseline	9 [8–10]	9 [8–10]	0.9	2 [2–3]	2 [2–3]	0.9	2 [2–3]	2 [2–3]	0.9	3.7 ± 0.9	4.0 ± 1.2	0.9	6.7 ± 1.5	7.1 ± 2.6	0.9	8.9 ± 1.7	9.5 ± 2.9	0.7
Week 2	9 [8–10]	9 [8–10]	0.9	2 [2–3]	2 [2–3]	0.9	2 [1–2]	2 [2–2]	0.7	3.1 ± 1.1	4.0 ± 1.3	0.008	11.9 ± 2.9	7.2 ± 2.5	<0.0001	15.2 ± 3.2	10.5 ± 2.6	<0.0001
Week 6	5 [4–7]	8 [8–9]	<0.0001	1 [1–2]	2 [2–3]	0.002	1 [1–1]	1 [1–2]	0.01	2.6 ± 0.9	3.9 ± 1.3	0.0006	15.3 ± 3.3	7.7 ± 2.5	<0.0001	19.0 ± 3.6	12.1 ± 2.8	<0.0001
Week 14	2 [1–3]	8 [8–9]	<0.0001	1 [0–2]	2 [2–3]	<0.0001	0 [0–1]	1 [1–2]	0.001	2.3 ± 0.6	3.9 ± 1.0	<0.0001	16.8 ± 3.5	7.7 ± 2.5	<0.0001	21.2 ± 3.7	11.7 ± 2.3	<0.0001
Significance level, baseline vs week 2	0.9	0.9	–	0.9	0.9	–	0.7	0.8	–	0.01	0.5	–	<0.0001	0.7	–	<0.0001	0.06	–

HC undergoing screening colonoscopy, devoid of any pathological findings, exhibited a BWT of 1.8 ± 0.03 mm, and patients with UC in remission presented with a BWT of 1.9 ± 0.05 mm. Statistical analysis revealed no significant difference [*p* > 0.05] between HC and UC patients in remission.

### 3.3. Dynamic, contrast-enhanced EUS [RT and TTP]

The inflamed colonic wall exhibited a notable increase in blood flow velocity. At baseline, RT was recorded at 6.7 ± 1.5 s. Within the initial 2 weeks of efficacious biological therapy, RT normalized significantly to 11.9 ± 2.9 s in cases of treatment success, while it remained unchanged at 7.2 ± 2.5 s in instances of treatment failure, resulting in a highly significant difference [*p* < 0.0001]. Furthermore, after 6 and 14 weeks of effective biological therapy, RT continued to decrease, reaching normal levels of 15.3 ± 3.3 and 16.8 ± 3.5 s, respectively. In contrast, RT in non-responding patients remained constant throughout the course of therapy [weeks 6 and 14 = 7.7 ± 2.5 s].

**Figure 3.**
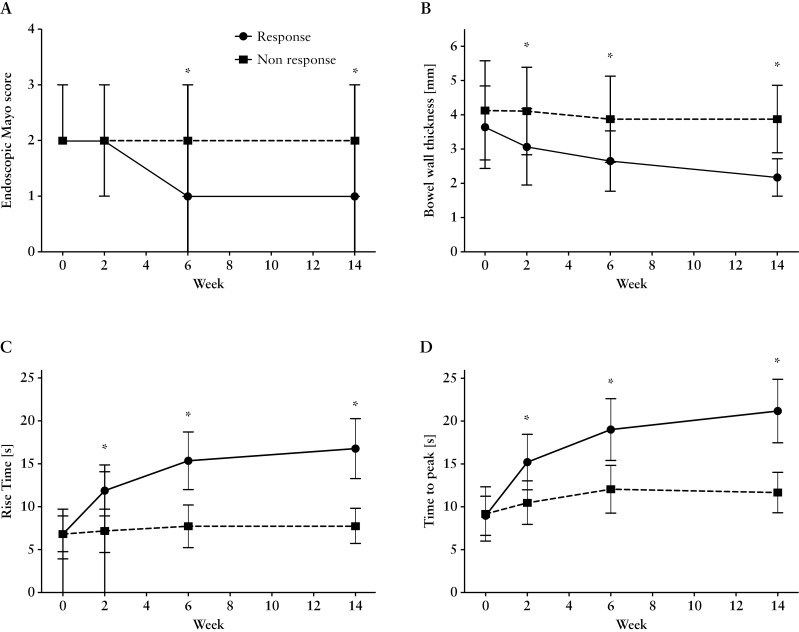
Comparison of endoscopic criteria between patients being responsive or non-responsive during 14 weeks of anti-TNF-alpha therapy. [A] Endoscopic Mayo score; [B] bowel wall thickness; [C] rise time; and [D] time to peak. Asterisks [*] indicate significant differences between the two groups at *p* < 0.05.

Similarly, TTP demonstrated parallel changes throughout the course of therapy. Commencing with a baseline TTP of 8.9 ± 1.7 s, TTP normalized significantly [*p* < 0.0001] within 2 weeks of therapy to 15.2 ± 3.2 s. Upon study completion after 14 weeks, a TTP of 21.2 ± 3.7 s was observed [*p* < 0.0001 compared to baseline levels]. In line with the changes observed in RT, no alterations in TTP were detected in patients who did not respond to biological therapy. Notably, significant differences between responders and non-responders in terms of TTP were discernible after only 2 weeks of therapy [*p* < 0.0001]. Comprehensive details of dCEUS parameters are documented in Table 2. In HC, the RT and TTP of the normal colon were 17.1 ± 1.3 and 20.8 ± 1.9 s, respectively. Comparable values were obtained for patients in remission [rUC] [Figure 3].

### 3.4. Laboratory findings

Markers of intestinal inflammation, including faecal calprotectin, and systemic inflammation, assessed through C-reactive protein [CRP] and leucocyte counts, exhibited comparable values at baseline in both study groups.

The concentration of faecal calprotectin was 491.5 ± 362.3 µg/g in patients prior to commencing biological therapy, and no discernible differences were observed between later-defined response and non-response groups. During the initial 2 weeks of biological therapy, regardless of whether ADA or IFX was administered, no significant alterations were noted in faecal calprotectin levels. However, after 6 weeks of therapy, a significant decrease in faecal calprotectin concentrations was observed in patients responding to anti-TNF therapy [faecal calprotectin ADA = 316.5 ± 293.6 µg/g; faecal calprotectin IFX = 208.1 ± 162.0 µg/g, *p* < 0.01]. Conversely, calprotectin levels remained unchanged in cases of non-response [*p* > 0.05 compared to baseline in both the ADA and IFX groups]. These differences in faecal calprotectin levels between responders and non-responders persisted throughout the subsequent 14-week treatment period.

Similar trends were noted in the levels of CRP and leucocyte concentrations across both therapy groups.

### 3.5. Endoscopic and total Mayo score

Prior to the commencement of anti-TNF therapy, patients presented with a median eMayo score of 2 [2–3], and no notable disparities were observed between the two distinct treatment regimens. During the initial 2 weeks of treatment, there were no discernible macroscopic changes in the mucosal surface when compared to baseline, irrespective of whether patients responded positively or not to therapy (Response: 2 [2–3]; Non-response: 2 [2–3]; *p* > 0.05). At 6 weeks, eMayo scores demonstrated a significant reduction in the group of patients who responded to therapy (week 6: eMayo = 1 [1–2], *p* < 0.0001 compared to baseline), while these scores remained unaltered in patients who did not respond to treatment (week 6: eMayo = 2 [2–3]; *p* > 0.05 compared to baseline). A marked difference was observed when comparing patients with a positive response to therapy versus those without a response after 6 weeks, encompassing both treatment regimens [*p* = 0.002]. It is noteworthy that, although patients responding to IFX exhibited a substantial decrease in the endoscopic Mayo score at week 6 when compared to baseline levels, a direct comparison between response and non-response in this subgroup at this specific time point failed to reach statistical significance [*p* = 0.06]. After 14 weeks, a significant discrepancy in the endoscopic Mayo scores was evident between patients with a therapeutic response and those without a response for both investigated medications [*p* < 0.0001 when comparing response vs non-response] [Table 2].

The total Mayo scores for the respective groups exhibited similar patterns. Once more, it was not feasible to distinguish between patients who were responsive to anti-TNF therapy and those who did not respond during the initial 2 weeks of treatment. Details are displayed in Table 2.

### 3.6. Nancy Histology Index

At baseline, patients scheduled for biological therapy presented with a mean Nancy Histology Index [NHI] of 2.1 ± 0.7 (NHI_ADA_ = 2.1 ± 0.7; NHI_IFX_ = 2.1 ± 0.6 [*p* > 0.05]). Patients who did not respond to therapy showed a marginal, though not significantly elevated level of histological inflammation with an index of 2.3 ± 0.5 compared to 1.9 ± 0.7 for the response group [*p* = 0.09].

After 2 weeks of treatment the NHI was not able to differentiate response to either of the two treatment options [Response group: NHI_total_ = 1.7 ± 0.6; Non-response group: NHI_total_ = 2.0 ± 0.5]. Though indices showed a trend towards improved levels of mucosal inflammation, this decline failed to reach statistical significance [*p* = 0.15]. At week 6 a significant difference between the histology indices became evident in case of response to biological therapy [Response: NHI_total_ = 0.9 ± 0.5; Non response: NHI_total_ = 1.5 ± 0.5; *p* = 0.0006].

At week 14 NHI values almost normalized in both response groups but remained significantly elevated in the non-response patients [Response: NHI_total_ = 0.5 ± 0.5; Non response: NHI_total_ = 1.4 ± 0.5; *p* < 0.0001]. A detailed overview of the histological inflammation levels is presented in Table 2.

### 3.7. Correlation analysis

At baseline assessment, a significant correlation was evident between the total and endoscopic Mayo scores and the NHI as well as the criteria assessed using EUS in all patients. When comparing the endoscopic Mayo score with NHI, a coefficient *R*^2^ of 0.28 was calculated [*p* = 0.002] in patients receiving biological therapy. Even more robust relationships were observed between NHI and the EUS criteria, with *p* = 0.0001 [NHI vs TWT *R*^2^ = 0.65; NHI vs RT *R*^2^ = 0.68; NHI vs TTP *R*^2^ = 0.65].

Of note, after 2 weeks of treatment, no correlation was observed between the endoscopic Mayo score and any of the respective EUS or histological criteria. In contrast, a strong correlation was established between NHI and BWT [*R*^2^ = 0.74], NHI and RT [*R*^2^ = 0.63], and NHI and TTP [*R*^2^ = 0.58] [*p* = 0.0001 for each comparison].

### 3.8. Calculation of cut-off levels

**Figure 4.**
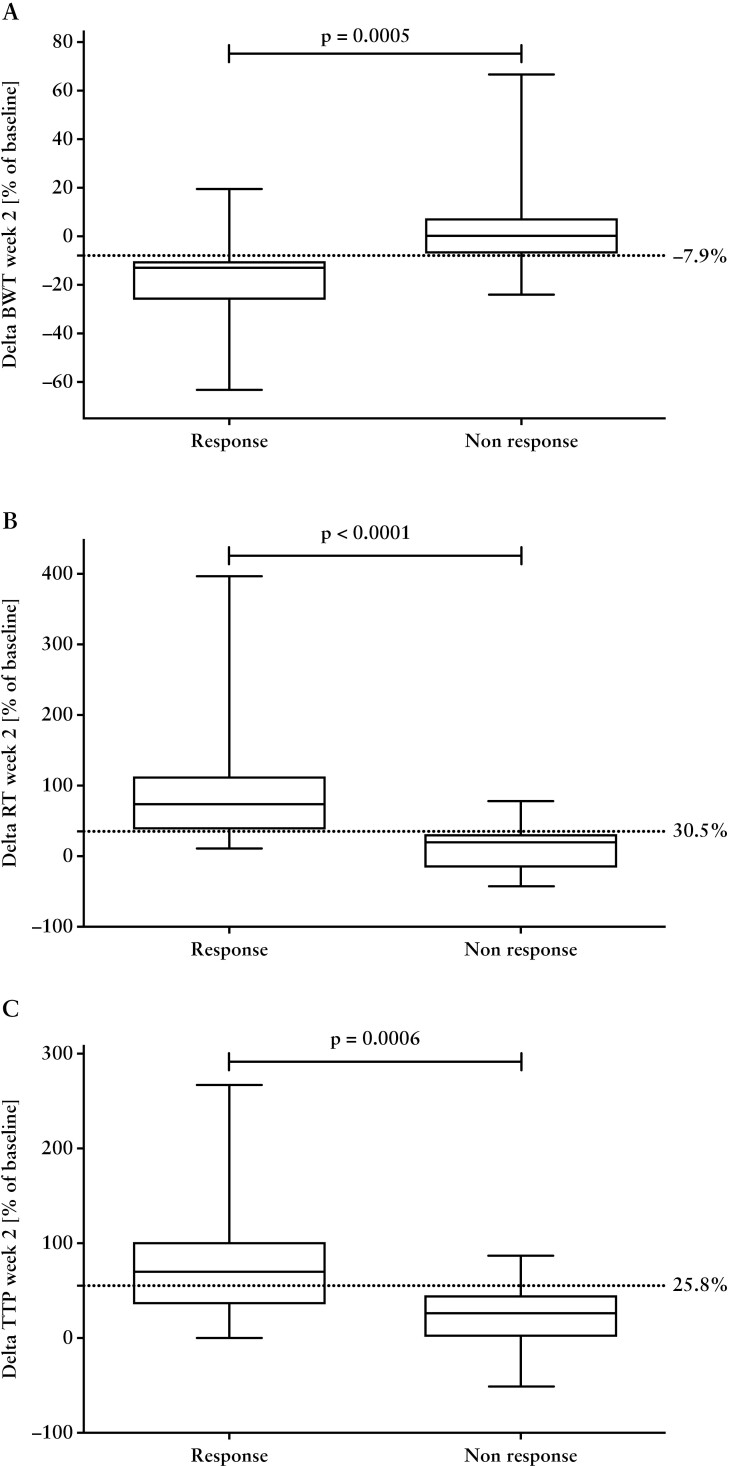
Relative changes of [A] bowel wall thickness [BWT], [B] rise time [RT], and [C] time to peak [TTP] in patients with response vs non-response to anti-TNF therapy at week 2. Cut-off values are indicated by dashed lines.

ROC analysis, along with the computation of Youden’s *J* index, identified a cut-off point of −7.9% of the initial BWT as a discriminative threshold to differentiate between patients with a treatment response and those without a response, after 2 weeks of therapy. This cut-off yielded a sensitivity of 0.9 (95% confidence interval [CI]: 0.58–0.99) and a specificity of 0.89 [95% CI: 0.67–0.98]. Furthermore, an increase of 30.5% in RT and 25.8% in TTP after 2 weeks of treatment was necessary to distinguish response from non-response patients. The corresponding sensitivities and specificities were 0.72 [95% CI: 0.39–0.94] and 0.95 [95% CI: 0.74–0.98], as well as 0.64 [95% CI: 0.31–0.89] and 1 [95% CI: 0.83–1], respectively [Figure 4].

### 3.9. Interobserver variability

Comparison of the eMayo score between two separate assessments conducted by experienced senior endoscopists yielded a weighted kappa coefficient of 0.75, indicative of substantial agreement. In contrast, when the eMayo score was evaluated by novices, the resulting weighted kappa coefficient was 0.58, signifying moderate agreement.

Assessment of the BWT by two senior endoscopists revealed a minor variation between their measurements, amounting to 4.8 ± 3.2%. Conversely, when novices conducted the evaluation, the difference in BWT relative to the ‘true’ results, as defined by the mean value obtained by the senior endoscopists, was more substantial, measuring 14.9 ± 10.4%.

Comparatively, the TTP and RT assessments demonstrated only slight differences between the senior endoscopists, with variances of 1.5 ± 1.4 and 3.5 ± 3.4%, respectively, while novices showed slightly higher differences in these measures.

### 3.10. Transabdominal, intestinal ultrasound

Regression analyses from the direct comparison revealed a statistically strong correlation between BWT assessments by tUS and dCEUS at distinct anatomical sites [sigmoid, rectosigmoid, and rectum] and time intervals [baseline and week 14] [*p* < 0.0001]. Notwithstanding the persistence of this correlation, there was a gradual decrease in the correlation coefficient *R*^2^ from the sigmoid [*r* = 0.92] to the rectosigmoid junction [*r* = 0.77] and ultimately to the rectum [*r* = 0.6].

**Figure 5.**
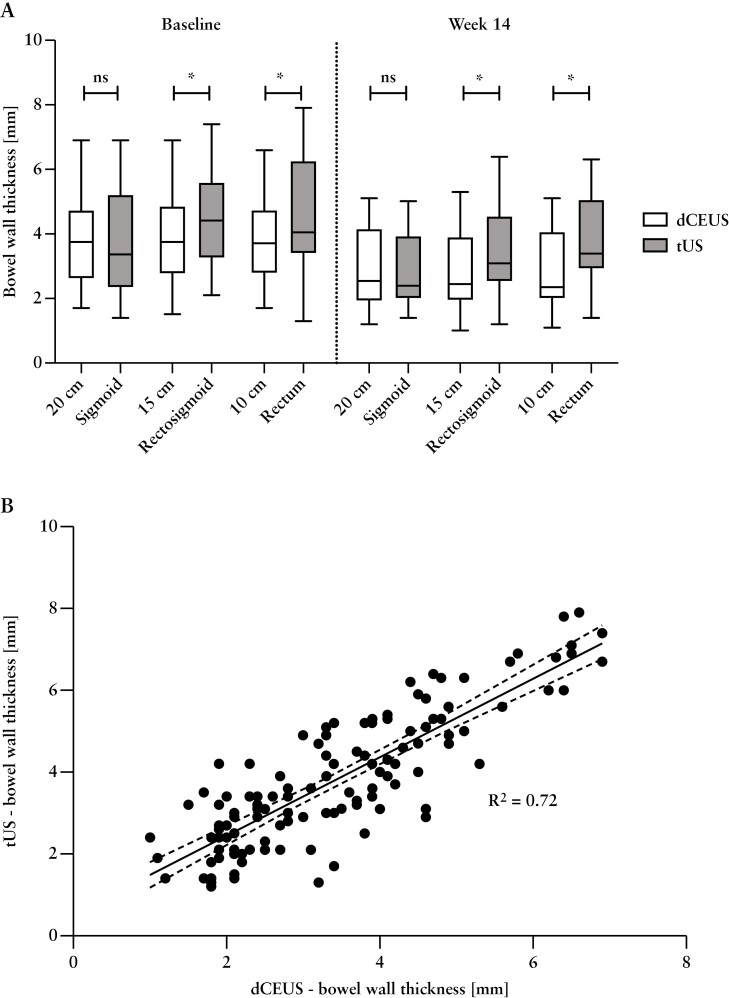
[A] Comparison of dCEUS and tUS between the different anatomical locations, baseline vs week 14. White boxes represent dCEUS examination, and grey boxes represent tUS examination. Asterisks [*] indicate significant differences between the two groups at *p* < 0.05. [B] Regression analysis of dCEUS compared to tUS in total, with all locations and times points assessed.

Upon paired comparison between the two diagnostic modalities at baseline, no significant difference was observed in BWT measurements for the sigmoid [BWT_dCEUS_ = 3.9 ± 1.6 mm; BWT_tUS_ = 3.8 ± 1.8 mm; *p* = 0.5]. Conversely, when comparing more distal anatomical locations [rectosigmoid junction and rectum], a significant disparity between the modalities emerged [rectosigmoid: BWT_dCEUS_ = 3.9 ± 1.5 mm; BWT_tUS_ = 4.5 ± 1.5 mm; *p* = 0.0003; rectum: BWT_dCEUS_ = 3.9 ± 1.5 mm; BWT_tUS_ = 4.6 ± 1.8 mm; *p* = 0.04]. A comprehensive overview of these differences at various locations and time points is presented in Table 3 and Figure 5. Based on the dCEUS values as reference we revealed a sensitivity of 0.45 [95% CI: 0.36–0.53] with a specificity of 0.61 [95% CI: 0.52–0.69] for the tUS assessment.

**Table 3.** Comparison of dCEUS and tUS between the different anatomical locations, baseline vs week 14.

Bowel wall thickness	Baseline			Week 14		
	dCEUS	tUS	*p*-value	dCEUS	tUS	*p*-value
Sigmoid [mm ± SD]	3.9 ± 1.6	3.8 ± 1.8	0.5	2.9 ± 1.2	2.9 ± 1.1	0.5
Rectosigmoid [mm ± SD]	3.9 ± 1.5	4.5 ± 1.5	0.0003	2.9 ± 1.2	3.4 ± 1.4	0.02
Rectum [mm ± SD]	3.9 ± 1.5	4.6 ± 1.8	0.04	2.9 ± 1.2	3.7 ± 1.4	0.002

### 3.11. Long-term follow-up

After 14 weeks of treatment, 45 patients transitioned into a long-term follow-up period, adhering to good clinical practice guidelines. Among the 29 patients who responded positively to anti-TNF therapy [yielding a response rate of 64.4%], they maintained their positive response at week 48, as evidenced by eMayo scores, BWT, TTP, and RT, which remained consistent with the levels observed at week 14. Consequently, it can be inferred that a long-term response extending to 48 weeks was predicted in nearly 90% of cases shortly after initiating biological therapy, within the initial 2 weeks.

The clinical follow-up extended for an average of 73.4 ± 11.4 weeks. Unchanged EUS criteria at week 2 served as an indicator of an elevated risk of long-term complications in patients who did not respond to the respective biological therapy. Such complications included hospitalization, which affected four individuals [constituting 8.9% of the total study population], as well as colectomy, which was performed in one patient [accounting for 2.2% of the study population].

## 4. Discussion

In this prospective longitudinal study, we evaluated the role of transmural healing in patients with UC using high-resolution and dynamic EUS. Our findings indicate that BWT, in conjunction with functional assessments of mucosal and submucosal blood flow, serve as highly sensitive early prognostic indicators after 2 weeks of treatment. These markers can effectively predict long-term therapeutic response and the likelihood of encountering prolonged therapeutic complications. It is noteworthy that the specified EUS criteria demonstrate superior performance when compared to the well-established scoring systems, specifically the total and endoscopic Mayo scores.

The STRIDE-II initiative outlined a set of recommended objectives for the management of UC. These include clinical criteria for the evaluation of short-term targets, intermediate treatment goals of normalizing inflammatory biomarkers, and long-term treatment objectives of achieving normal quality of life and attaining endoscopic remission. Notably, histological and transmural healing were not suggested as primary treatment goals.^3^ In a recent international Delphi consensus, a set of recommendations for defining comprehensive disease control as a treatment target in UC was formulated incorporating additional parameters derived from patient-reported outcomes such as bowel urgency, abdominal pain, extraintestinal manifestations, fatigue, and sleep disturbance. In parallel with the STRIDE-II framework, the expert panel reached a consensus to include endoscopic remission in their recommendations, but only as a secondary target.^18^ This adjustment was based on inconsistent data on the long-term outcomes associated with different thresholds of the endoscopic Mayo score [specifically, 0 vs ≤1].^19–21^

These recommendations are in concordance with our observations regarding the limited utility of the total and endoscopic Mayo scores in predicting early therapy response as well as long-term outcomes, which is attributed to two primary drawbacks associated with the scoring systems: [i] significant interobserver variability^7,22^ and [ii] delayed responsiveness to therapy. Of note, the endoscopic Mayo score, which is commonly used for assessing mucosal healing, is validated only after a minimum of 8 weeks of treatment and does not discern differences during the initial weeks of therapy.^8,23^ Our current data substantiate these conclusions, as we did not detect any alterations in the endoscopic Mayo score during the initial 2 weeks of treatment, and only slight differences between responders and non-responders to biological therapy emerged at week 6.

### 4.1. Bowel wall thickness

EUS assessment is a valuable technique, particularly in the context of the lower gastrointestinal tract, where it has primarily been established for the staging of rectal cancer.^24^ In earlier investigations conducted by our group, the utility of rectal EUS was explored for distinguishing between CD, UC, and HC. By amalgamating EUS criteria including BWT and the presence of paracolonic lymph nodes, we achieved a 92.3% sensitivity in discriminating active CD from UC. BWT in the recto-sigmoid colon exhibited a robust correlation with histological disease activity prior to the commencement of anti-inflammatory therapy,^12^ which has been validated by the baseline data of the current study.

In the context of anti-TNF therapy response, our investigation revealed that BWT experienced a notable reduction within the initial 2 weeks, preceding changes in the superficial endoscopic appearance by several weeks. The calculated cut-off value for predicting therapy response at this very early stage was determined to be a 7.9% reduction in BWT, with a sensitivity and specificity of 0.9. Previous investigations have shown a robust association between BWT and disease activity among individuals with IBD visualized by a variety of imaging modalities, including computed tomography, magnetic resonance imaging, scintigraphy, and tUS.^25^ tUS studies have emphasized the utility of BWT as a reliable criterion for quantifying disease activity in CD. The diagnostic sensitivity and specificity of BWT for CD have been reported to range from 63 to 100% and from 77 to 100%, respectively.^26^ Of note, the sensitivity of diagnostic methods varies significantly across different anatomical regions. US demonstrates its highest accuracy in the sigmoid and descending colon with a notably low sensitivity being reported for the rectum (14.9% [95% CI 6.8–23.0]).^27^

A head-to-head comparison conducted in our study demonstrated a compromised overall sensitivity of 0.45, accompanied by a corresponding specificity of 0.61 for the measurement of BWT using tUS, which are in line with the findings mentioned above. The current data also indicate that the depiction of distal intestinal segments is associated with a higher standard deviation and significantly different values compared to endoluminal imaging. This is probably attributed to an increasing tangential scanning plane in the deep pelvic regions. This issue can be circumvented by the consistently orthogonal scanning plane of transrectal ultrasound.

As an alternative, perineal ultrasound [PNUS] has the potential to address the limitations of tUS in assessing pathologies of the distal rectum. This non-invasive technique, although not widely adopted, has been the subject of limited research. In a recent study involving patients with UC, PNUS emerged as an independent predictor of both endoscopic and histological healing when BWT was <4 mm. This suggests its potential utility in guiding UC therapy. However, the lack of longitudinal data acquisition and the absence of precise standard deviations in reported findings may impose limitations of the method. Furthermore, given an ultrasound probe infiltration depth of ~5 cm, PNUS is primarily suitable for assessing the very distal rectum, with the drawback of a tangential scanning plane when examining more proximal anatomical areas, as previously discussed.^28,29^

A prospective, multicentre study, known as the TRUST trial, investigated the role of transmural response and transmural healing, assessed using intestinal tUS in patients with IBD. Within a 12-week treatment period, nearly 66% of CD patients and 77% of UC patients exhibited transmural response. Notably, a long-term follow-up at 52 weeks revealed favourable outcomes in patients who had previously demonstrated transmural healing at week 12.^30^

While these findings are consistent with our present data, we believe that rectal EUS offers significant advantages over tUS and PNUS in the evaluation of disease activity: [i] superior imaging resolution by directly contacting inflamed areas in a perpendicular plane, and [ii] direct access to the rectal surface in combination with colonoscopy [one-stop-shop examination].

### 4.2. Dynamic contrast enhanced endoscopic ultrasound

Enhanced vascularization represents a characteristic hallmark of intestinal inflammation, yet its precise quantification remains a challenge. So far, the Limberg score, as a four graded semi-quantitative tool, has primarily been used to assess intestinal blood flow but does not offer detailed visualization of the microcirculation within the mucosa and submucosa.^31^ Guidelines from the European Federation of Societies for Ultrasound in Medicine and Biology [EFSUMB] have endorsed the use of CEUS for estimating disease activity in patients with IBD. CEUS has the unique capability to visualize bowel wall vascularity at the microcirculatory level.^32^ Studies have demonstrated that time–signal intensity curves following bolus contrast injection exhibit a strong correlation with endoscopic and histological disease activity in IBD patients.^33,34^ These findings are in alignment with our current observations involving dCEUS in patients with active UC at baseline. We identified a strong correlation between the NHI, the endoscopic Mayo score, and the evaluation of RT and TTP at baseline. By utilizing signal intensity curves, we were able to distinguish patients who responded to anti-TNF therapy from those who did not, within the initial 2 weeks, with calculated cut-off values of a 30% increase in RT and a 26% increase in TTP.

Following 12 weeks of pharmacological treatment, Quaia *et al*. observed a comparable normalization of the time–intensity curves among CD responders.^35^ Saevik *et al*. assessed contrast-enhanced tUS in CD patients receiving anti-inflammatory treatment, either through systemic steroids or anti-TNF therapy. Following 4 weeks of treatment, they also observed a significant normalization in peak enhancement among patients who responded to their respective therapies. In our evaluation of UC patients with EUS, we obtained analogous results and additionally demonstrated the ability to predict therapy response using the previously mentioned cut-off levels.^36^

Our findings indicate that the early response to anti-TNF therapy is characterized by the restoration of microvascular blood flow as an initial step of healing. Subsequently, there is a decrease in the presence of inflammatory cells within the mucosa and submucosa, ultimately culminating in a reduction of the endoscopic Mayo score. This hypothesis aligns with pathophysiological insights gained from patients with IBD.^37,38^

In contrast to widely employed endoscopic scoring systems, which exhibit interobserver variabilities with weighted Kappa coefficients ranging from 0.44 to 0.76, depending on the level of experience, quantitative parameters of colonic inflammation, specifically BWT, TTP, and RT, demonstrate high reproducibility, even with limited experience in rectal EUS. Therefore, EUS may emerge as a dependable tool for quantifying inflammation in IBD and assessing therapy response. The potential for employing artificial intelligence algorithms presents an exciting opportunity to further automate assessment in these cases.

#### 4.2.1. Limitations

Despite the compelling results achieved in predicting early therapy response through EUS criteria, it is imperative to acknowledge certain limitations of this study. [i] The sample size, with a total of 45 patients completing the study, is relatively small, particularly in the context of the IFX group. [ii] CEUS is costly and not commonly utilized, especially in private practices, and thus is restricted to specialized centres with a substantial caseload of IBD patients. [iii] Although only sigmoidoscopy is required at various time points, EUS is an invasive procedure, which can be uncomfortable for patients and carries the potential for complications, such as bleeding, perforation, or allergic reactions to the contrast agent.

Ultimately, an imaging score incorporating tUS, PNUS and rectal EUS may emerge as the preferred diagnostic tool for tailoring therapeutic strategies in patients with IBD.

## 5. Conclusions

While colonoscopy remains the gold standard for assessing inflammation in UC, our study highlights the novel application of EUS for precise quantification of BWT and real-time vascularity dynamics in UC. EUS changes emerge as early indicators, greatly preceding mucosal healing. By combining measurements of BWT with CEUS, we introduce a potential marker for early therapeutic response during anti-TNF therapy, which may also serve as an indicator of non-response. These findings open promising avenues for more effective and timely monitoring of treatment outcomes in patients with UC.

## Supplementary Data

Supplementary data are available online at *ECCO-JCC* online.

jjae034_suppl_Supplementary_Material

## Data Availability

The data underlying this article will be shared on reasonable request to the corresponding author.

## References

[1] Soriano CR , Powell CR, Chiorean MV, Simianu VV. Role of hospitalization for inflammatory bowel disease in the post-biologic era. World J Clin Cases 2021;9:7632–42. 34621815 10.12998/wjcc.v9.i26.7632 PMC8462259

[2] Peyrin-Biroulet L , Sandborn W, Sands BE, et al. Selecting Therapeutic Targets in Inflammatory Bowel Disease (STRIDE): determining therapeutic goals for treat-to-target. Am J Gastroenterol 2015;110:1324–38. 26303131 10.1038/ajg.2015.233

[3] Turner D , Ricciuto A, Lewis A, et al; International Organization for the Study of IBD. STRIDE-II: an update on the Selecting Therapeutic Targets in Inflammatory Bowel Disease (STRIDE) Initiative of the International Organization for the Study of IBD (IOIBD): determining therapeutic goals for treat-to-target strategies in IBD. Gastroenterology 2021;160:1570–83. 33359090 10.1053/j.gastro.2020.12.031

[4] Colombel JF , Rutgeerts P, Reinisch W, et al. Early mucosal healing with infliximab is associated with improved long-term clinical outcomes in ulcerative colitis. Gastroenterology 2011;141:1194–201. 21723220 10.1053/j.gastro.2011.06.054

[5] Lichtenstein GR , Rutgeerts P. Importance of mucosal healing in ulcerative colitis. Inflamm Bowel Dis 2010;16:338–46. 19637362 10.1002/ibd.20997

[6] Rutgeerts P , Vermeire S, Van Assche G. Mucosal healing in inflammatory bowel disease: impossible ideal or therapeutic target? Gut 2007;56:453–5. 17369375 10.1136/gut.2005.088732 PMC1856849

[7] Daperno M , Comberlato M, Bossa F, et al. Inter-observer agreement in endoscopic scoring systems: preliminary report of an ongoing study from the Italian Group for Inflammatory Bowel Disease (IG-IBD). Dig Liver Dis 2014;46:969–73. 25154049 10.1016/j.dld.2014.07.010

[8] Walsh AJ , Ghosh A, Brain AO, et al. Comparing disease activity indices in ulcerative colitis. J Crohns Colitis 2014;8:318–25. 24120021 10.1016/j.crohns.2013.09.010

[9] Mohammed Vashist N , Samaan M, Mosli MH, et al. Endoscopic scoring indices for evaluation of disease activity in ulcerative colitis. Cochrane Database Syst Rev 2018;1:CD011450. 29338066 10.1002/14651858.CD011450.pub2 PMC6491285

[10] Tontini GE , Bisschops R, Neumann H. Endoscopic scoring systems for inflammatory bowel disease: pros and cons. Expert Rev Gastroenterol Hepatol 2014;8:543–54. 24650249 10.1586/17474124.2014.899899

[11] Piazza OSN , Noviello D, Filippi E, et al. Superior predictive value of transmural over endoscopic severity for colectomy risk in ulcerative colitis: a multicenter prospective cohort study. J Crohns Colitis 2023;26:jjad152. doi:10.1093/ecco-jcc/jjad152 PMC10896635 37632350

[12] Ellrichmann M , Wietzke-Braun P, Dhar S, et al. Endoscopic ultrasound of the colon for the differentiation of Crohn’s disease and ulcerative colitis in comparison with healthy controls. Aliment Pharmacol Ther 2014;39:823–33. 24612000 10.1111/apt.12671

[13] Shimizu S , Tada M, Kawai K. Value of endoscopic ultrasonography in the assessment of inflammatory bowel diseases. Endoscopy 1992;24:354–8. 1633780 10.1055/s-2007-1010499

[14] Schroeder KW , Tremaine WJ, Ilstrup DM. Coated oral 5-aminosalicylic acid therapy for mildly to moderately active ulcerative colitis. A randomized study. N Engl J Med 1987;317:1625–9. 3317057 10.1056/NEJM198712243172603

[15] Riphaus A , Wehrmann T, Hausmann J, et al. Update S3-guideline: “sedation for gastrointestinal endoscopy” 2014 (AWMF-register-no. 021/014). Z Gastroenterol 2016;54:58–95. 26751118 10.1055/s-0041-109680

[16] Greis C. Quantitative evaluation of microvascular blood flow by contrast-enhanced ultrasound (CEUS). Clin Hemorheol Microcirc 2011;49:137–49. 22214685 10.3233/CH-2011-1464

[17] Marchal-Bressenot A , Salleron J, Boulagnon-Rombi C, et al. Development and validation of the Nancy histological index for UC. Gut 2017;66:43–9. 26464414 10.1136/gutjnl-2015-310187

[18] Schreiber S , Danese S, Dignass A, et al. Defining comprehensive disease control for use as a treatment target for ulcerative colitis in clinical practice: International Delphi Consensus Recommendations. J Crohns Colitis 2024;18:91–105. 37586038 10.1093/ecco-jcc/jjad130 PMC10821705

[19] Barreiro-de Acosta M , Vallejo N, de la Iglesia D, et al. Evaluation of the risk of relapse in ulcerative colitis according to the degree of mucosal healing (Mayo 0 vs 1): a longitudinal cohort study. J Crohns Colitis 2016;10:13–9. 26351390 10.1093/ecco-jcc/jjv158

[20] Boal Carvalho P , Dias de Castro F, Rosa B, Moreira MJ, Cotter J. Mucosal healing in ulcerative colitis--when zero is better. J Crohns Colitis 2016;10:20–5. 26438714 10.1093/ecco-jcc/jjv180

[21] Lee SD , Allegretti JR, Steinwurz F, et al. Tofacitinib as a maintenance therapy in patients with ulcerative colitis stratified by OCTAVE Sustain baseline Mayo endoscopic subscore. BMC Gastroenterol 2023;23:34. 36755231 10.1186/s12876-022-02508-2 PMC9906955

[22] Daperno M , Comberlato M, Bossa F, et al; IGIBDEndo Group. Training programs on endoscopic scoring systems for inflammatory bowel disease lead to a significant increase in interobserver agreement among community gastroenterologists. J Crohns Colitis 2017;11:556–61. 28453758 10.1093/ecco-jcc/jjw181

[23] Feagan BG , Sandborn WJ, D’Haens G, et al. The role of centralized reading of endoscopy in a randomized controlled trial of mesalamine for ulcerative colitis. Gastroenterology 2013;145:149–57.e2. 23528626 10.1053/j.gastro.2013.03.025

[24] Ghoneem E , Shabana ASA, El Sherbini M, et al. Endoluminal ultrasound versus magnetic resonance imaging in assessment of rectal cancer after neoadjuvant therapy. BMC Gastroenterol 2022;22:542. 36575373 10.1186/s12876-022-02628-9 PMC9793528

[25] Horsthuis K , Bipat S, Bennink RJ, Stoker J. Inflammatory bowel disease diagnosed with US, MR, scintigraphy, and CT: meta-analysis of prospective studies. Radiology 2008;247:64–79. 18372465 10.1148/radiol.2471070611

[26] Alshammari MT , Stevenson R, Abdul-Aema B, et al. Diagnostic accuracy of non-invasive imaging for detection of colonic inflammation in patients with inflammatory bowel disease: a systematic review and meta-analysis. Diagnostics (Basel) 2021;11:1926. 34679624 10.3390/diagnostics11101926 PMC8534724

[27] Parente F , Greco S, Molteni M, et al. Role of early ultrasound in detecting inflammatory intestinal disorders and identifying their anatomical location within the bowel. Aliment Pharmacol Ther 2003;18:1009–16. 14616167 10.1046/j.1365-2036.2003.01796.x

[28] Nuernberg D , Saftoiu A, Barreiros AP, et al. EFSUMB recommendations for gastrointestinal ultrasound part 3: endorectal, endoanal and perineal ultrasound. Ultrasound Int Open. 2019;5:E34–51. 30729231 10.1055/a-0825-6708 PMC6363590

[29] Sagami S , Kobayashi T, Aihara K, et al. Transperineal ultrasound predicts endoscopic and histological healing in ulcerative colitis. Aliment Pharmacol Ther 2020;51:1373–83. 32383166 10.1111/apt.15767

[30] Esteban JM , Maldonado L, Sanchiz V, Minguez M, Benages A. Activity of Crohn’s disease assessed by colour Doppler ultrasound analysis of the affected loops. Eur Radiol 2001;11:1423–8. 11519551 10.1007/s003300000770

[31] Limberg B , Osswald B. Diagnosis and differential diagnosis of ulcerative colitis and Crohn’s disease by hydrocolonic sonography. Am J Gastroenterol 1994;89:1051–7. 8017364

[32] Sidhu PS , Cantisani V, Dietrich CF, et al. The EFSUMB guidelines and recommendations for the Clinical Practice of Contrast-Enhanced Ultrasound (CEUS) in non-hepatic applications: update 2017 (Short Version). Ultraschall Med 2018;39:154–80. 29510440 10.1055/s-0044-101254

[33] Medellin-Kowalewski A , Wilkens R, Wilson A, Ruan J, Wilson SR. Quantitative contrast-enhanced ultrasound parameters in Crohn disease: their role in disease activity determination with ultrasound. Am J Roentgenol 2016;206:64–73. 26700336 10.2214/AJR.15.14506

[34] Ponorac S , Dahmane Gosnak R, Urlep D, Kljucevsek D. Diagnostic value of quantitative contrast-enhanced ultrasound in comparison to endoscopy in children with Crohn’s disease. J Ultrasound Med 2023;42:193–200. 35748308 10.1002/jum.16044

[35] Quaia E , Cabibbo B, De Paoli L, Toscano W, Poillucci G, Cova MA. The value of time–intensity curves obtained after microbubble contrast agent injection to discriminate responders from non-responders to anti-inflammatory medication among patients with Crohn’s disease. Eur Radiol 2013;23:1650–9. 23306710 10.1007/s00330-012-2754-1

[36] Saevik F , Nylund K, Hausken T, Odegaard S, Gilja OH. Bowel perfusion measured with dynamic contrast-enhanced ultrasound predicts treatment outcome in patients with Crohn’s disease. Inflamm Bowel Dis 2014;20:2029–37. 25185684 10.1097/MIB.0000000000000159 PMC4213134

[37] Principi M , Scicchitano P, Carparelli S, et al. Influence of systemic manifestations of inflammatory bowel diseases on endothelial function and cardiovascular risk. Minerva Med 2022;113:291–9. 33913656 10.23736/S0026-4806.21.06970-6

[38] Cibor D , Domagala-Rodacka R, Rodacki T, Jurczyszyn A, Mach T, Owczarek D. Endothelial dysfunction in inflammatory bowel diseases: pathogenesis, assessment and implications. World J Gastroenterol 2016;22:1067–77. 26811647 10.3748/wjg.v22.i3.1067 PMC4716020

