# Supplementary material for: Contrast-Enhanced Endoscopic Ultrasound Detects Early Therapy Response Following Anti-TNF Therapy in Patients with Ulcerative Colitis
Source: J Crohns Colitis. 2024 Mar 8;18(7):1012–24. doi: 10.1093/ecco-jcc/jjae034 (PMC11302966; doi:10.1093/ecco-jcc/jjae034)
Supplement: jjae034_suppl_Supplementary_Material [file jjae034_suppl_supplementary_material.docx]

**Supplement 1: Additional inclusion and exclusion criteria**

| **Inclusion criteria:** |
| --- |
| -       indication for ADA or IFX therapy, assessed by an independent gastroenterologist |
| -       no TNF antagonists within 60 days before enrollment or cyclosporine, thalidomide, or investigational drugs within 30 days before enrollment |
| -       written informed consent |
| -       ASA classification (American Society of Anaesthesiology) I - II |
| **Exclusion criteria:** |
| -       known or suspected hypersensitivity to contrast agent SonoVue and/or butylscopolamin |
| -       gastrointestinal obstruction or suspected perforation |
| -       serious cardiovascular, renal or hepatic disease |
| -       impaired coagulation |
| -       elevated serum creatinine |
| -       acute diverticulitis |
| -       concomitant viral colitis i.e. cytomegalovirus infection |
| -       NSAID use and use of any medication that could potentially cause or aggravate colitis during the last four weeks prior initiation of biological therapy |
| -       toxic megacolon |
| -       abdominal abscess |
| -       history of colonic surgery, escpeially colectomy |
| -       increased risk of infectious complications |
| (e.g. pyogenic infection <4weeks, enteric pathogens detected on stool analysis, active or latent tuberculosis, immunodeficiency, hepatitis B or C, live vaccination <4weeks), |
| -       pregnancy or lactation, |
| -       colonic dysplasia or adenomas |
| -       malignant neoplasms. |
